# Anti-drude metal of bosons

**DOI:** 10.1038/s41467-022-29708-4

**Published:** 2022-04-19

**Authors:** Guido Masella, Nikolay V. Prokof’ev, Guido Pupillo

**Affiliations:** 1 University of Strasbourg, CNRS, CESQ & ISIS (UMR 7006), 67000 Strasbourg, France; 2 Department of Physics, University of Massachusetts, Amherst, 01003 Massachusetts, MA USA

**Keywords:** Quantum fluids and solids

## Abstract

In the absence of frustration, interacting bosons in their ground state in one or two dimensions exist either in the superfluid or insulating phases. Superfluidity corresponds to frictionless flow of the matter field, and in optical conductivity is revealed through a distinct *δ*-functional peak at zero frequency with the amplitude known as the Drude weight. This characteristic low-frequency feature is instead absent in insulating phases, defined by zero static optical conductivity. Here we demonstrate that bosonic particles in disordered one dimensional chains can also exist in a conducting, non-superfluid, phase when their hopping is of the dipolar type, often viewed as short-ranged in one dimension. This phase is characterized by finite static optical conductivity, followed by a broad anti-Drude peak at finite frequencies. Off-diagonal correlations are also unconventional: they feature an integrable algebraic decay for arbitrarily large values of disorder. These results do not fit the description of any known quantum phase, and strongly suggest the existence of an unusual conducting state of bosonic matter in the ground state.

## Abstract (web summary)

Interacting bosons in one or two dimensions are expected to be in either a superfluid or an insulating ground state. Here, the authors show numerically that an experimentally relevant 1D model of disordered bosons with dipolar couplings supports an unusual metallic phase that does not fit into existing descriptions.

## Introduction

Quantum phases of matter are distinguished by their static and dynamical properties, quantified by correlation functions. For interacting bosonic matter in one dimension, the superfluid phase is characterized by a non-integrable algebraic decay of static one-body (off-diagonal) correlations as a function of distance and by a *δ*-functional peak at zero frequency in the optical conductivity, respectively. The latter is reflecting a singular response to a weak externally applied field. A strong enough disorder can induce a quantum phase transition from the superfluid to an insulating phase, known as the Bose glass^1^. In this phase, off-diagonal correlations decay exponentially with distance and the optical conductivity starts from zero at zero frequency, reflecting the absence of long-lived collective modes at low energy. These two phases exhaust the known possibilities for disordered bosons in one dimension in the absence of frustration, whereby frustration we understand a situation when the path-integral representation of quantum statistics in imaginary time is not sign-positive. In this work, we provide numerical evidence for the existence, in one-dimensional (*d* = 1) lattice systems, of a disorder-induced phase that is neither superfluid nor insulating. Despite featuring an algebraic decay of off-diagonal correlations, it has zero superfluid density and its optical conductivity is finite at zero frequency. The latter is followed by a broad peak at a finite frequency of the order of the nearest-neighbor hopping energy. Because of this characteristic “anti-Drude” behavior of optical conductivity, with finite minimum instead of maximum at zero frequency, we term this phase an anti-Drude metal of bosons (aDMB).

The aDMB phase is a result of the interplay between interactions, disorder, and particle hopping, which we choose to be of the dipolar type. The latter is usually considered as short-ranged in *d* = 1^2^. For non-interacting models with short-range hopping, the disorder is generally expected to localize all wave functions exponentially (Anderson localization)^3^. However, recent theoretical works have demonstrated that single-particle states can localize algebraically in the presence of couplings that decay with distance as a power-law^4–8^. What happens in strongly interacting systems remained an open question, and this work provides the first answers with the discovery of the aDMB ground state.

Dipolar couplings have been already experimentally realized for internal excitations of cold magnetic atoms^9–14^, Rydberg excited atoms^15–17^, ions^18,19^, and molecules^20^. The propagation of excitations with dipolar couplings in the presence of the disorder is also highly relevant for a variety of solid-state systems, including nuclear spins^21^, nitrogen-vacancy centers in diamonds^22^, or two-level emitters placed near a photonic crystal waveguide^23^.

We note that the existence of a metallic bosonic phase has been suggested previously^24–26^; e.g., in the context of finite-temperature strange metal behavior of high-temperature superconductors^25,27^ and as a possible ground state in lattice models with multi-particle interactions^26,28,29^. However, up to date, the existence of a metallic phase of bosons has not been confirmed by exact methods in any physical system. Since frustrated spin systems featuring a variety of spin-liquid phases can be always reformulated in terms of strongly interacting bosons, we exclude frustrated models from this discussion.

## Results

We consider the following Hamiltonian for hard-core bosons confined to one-dimensional lattices

1 $${{{{{{{\mathcal{H}}}}}}}}=-t\mathop{\sum}\limits_{i < j}\frac{{a}^{3}}{| {r}_{ij}{| }^{3}}\left[{b}_{i}^{{{{\dagger}}} }{b}_{j}+{{{{{{{\rm{H}}}}}}}}.{{{{{{{\rm{c}}}}}}}}.\right]+\mathop{\sum}\limits_{i}{\epsilon }_{i}{n}_{i},({n}_{i}\le 1).$$

We employ standard notations for bosonic creation and annihilation operators on site *i* and occupation numbers, ${n}_{i}={b}_{i}^{{{{\dagger}}} }{b}_{i}$, that cannot exceed unity in the allowed Fock states. The nearest-neighbor hopping amplitude, *t*, and the lattice spacing, *a*, are taken as units of energy and length, respectively. Hopping amplitudes between sites *i* and *j* decay with the distance between them as ${r}_{ij}^{-3}$, and *ϵ*_*i*_ are random on-site energies uniformly distributed between −*W* and *W*. In spin language, Eq. (1) is equivalent to an XY Hamiltonian with dipolar couplings, which, in the absence of disorder, can be realized in experiments with cold polar molecules^20^, trapped ions^18,19^, and Rydberg atoms^15,16,30^ (the latter can also be disordered^17^). Recent theoretical works provide strong evidence that Eq. (1) supports a many-body localized (MBL) phase at finite energy^20,31–34^. Our result then implies that the MBL transition out of aDMB takes place as the temperature is increased. In a system with an upper bound on the maximal energy per particle, this result is not that surprising^35^.

In the following, we determine the ground-state quantum phases of Eq. (1) using large-scale path-integral quantum Monte Carlo simulations based on the Worm algorithm^36^. Without loss of generality, we focus on the particle density *ρ* = 1/2.

For nearest-neighbor hopping only, one-dimensional hard-core bosons behave as spinless fermions and bosonic exchange has to involve all particles in the liquid. A regular system would have finite superfluid density, *ρ*_s_, that characterizes the response to a twisted boundary condition caused by an external vector potential field. It can be conveniently computed by quantum Monte Carlo methods, see Methods, through the statistics of winding numbers, ${{{{{{{\mathcal{W}}}}}}}}$, using the Pollock–Ceperley relation ${\rho }_{{{{{{{{\rm{s}}}}}}}}}\propto {\langle {{{{{{{\mathcal{W}}}}}}}}\rangle }^{2}$^37^ (see Methods). However, it is well known that the superfluid density of this system is immediately suppressed by any finite strength of disorder *W*, due to Anderson localization^1^. Dipolar hopping changes this picture entirely, by allowing for pair-wise bosonic exchanges, somewhat similar to soft-core particles. One then expects superfluidity to be robust against weak disorder, and, possibly, undergo a quantum phase transition to a non-superfluid phase when disorder exceeds some critical value *W*_c_.

**Fig. 1:**
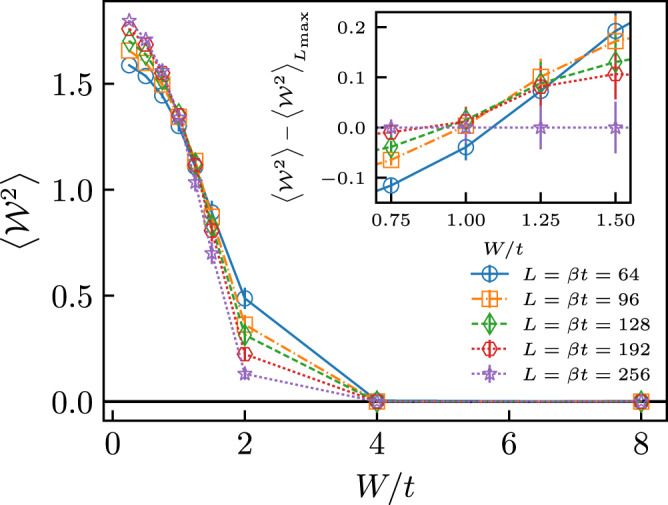
Mean-square winding numbers. $\langle {{{{{{{{\mathcal{W}}}}}}}}}^{2}\rangle$ as a function of the disorder strength *W* for lattice sizes *L* = 64 (blue circles), 96 (orange squares), 128 (green diamonds), 192 (red hexagons), 256 (purple stars). Inset highlights the area near the phase transition, showing crossing points between the curves within the interval *W*_*c*_ = 1.00 ± 0.15; the curve corresponding to the largest size (*L* = 256) is subtracted from all data for clarity. Vertical error bars indicate the estimated uncertainty from the Monte Carlo simulations and disorder-averages.

Figure 1 shows numerical results for the mean-square winding number $\langle {{{{{{{{\mathcal{W}}}}}}}}}^{2}\rangle$ as a function of the disorder strength *W* for different lattice sizes *L*. Mean-square winding number is expected to be scale-invariant at a continuous phase transition, regardless of the system dimension. This allows one to identify the critical disorder strength *W*_*c*_ where superfluidity is lost by the crossing point of the $\langle {{{{{{{{\mathcal{W}}}}}}}}}^{2}\rangle$-vs-*W* curves for different values of *L*. Figure 1 shows that all sizes larger than *L* > 64 cross at *W*_c_ = 1.00 ± 0.15 (see Inset), signaling the transition from a superfluid phase for *W* < *W*_c_ to a quantum phase that is not superfluid for *W* > *W*_c_. In the following, we focus on characterizing the properties of this non-superfluid phase with *W* > *W*_c_ by studying its correlation functions and optical conductivity.

**Fig. 2:**
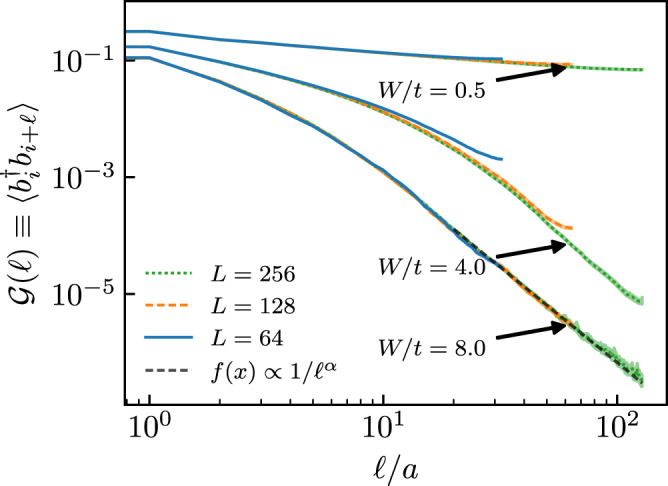
Decay of the disorder-averaged one-body density matrix. ${{{{{{{\mathcal{G}}}}}}}}(\ell )$ as a function of distance *ℓ* for system sizes *L* = 64 (blue solid lines), 128 (yellow dashed lines), and 256 (green dotted lines), and values of the disorder strength *W* = 0.5, 4.0, and 8.0 (top to bottom). Data were shown on the doubly logarithmic scale. The gray dashed line corresponds to a power-law fit 1/*ℓ*^*α*^ with *α* = 3.26(2) of the large distance behavior for *L* = 256 and *W* = 8.0. Vertical error bars indicate the estimated uncertainty from the Monte Carlo simulations and disorder-averages.

The one-body density matrix ${{{{{{{\mathcal{G}}}}}}}}(\ell )=\langle {b}_{i}^{{{{\dagger}}} }{b}_{i+\ell }\rangle$ is expected to show a non-integrable algebraic decay with the distance *ℓ* for a one-dimensional superfluid ground state, while in an insulating phase it is expected to decay exponentially, e.g. in a crystalline phase or a Bose glass. Figure 2 shows ${{{{{{{\mathcal{G}}}}}}}}(\ell )$ for the Hamiltonian Eq. (1), for chosen values of the disorder strength *W*. In the superfluid phase with *W* = 0.5 < *W*_c_, we observe a slow algebraic decay of ${{{{{{{\mathcal{G}}}}}}}}(\ell )$, as expected. We find that an initial exponential decay of ${{{{{{{\mathcal{G}}}}}}}}(\ell )$ is followed at large distances *ℓ* by an algebraic decay in the non-superfluid phase for *W* > *W*_c_ that is well described by the power-law dependence ${{{{{{{\mathcal{G}}}}}}}}(\ell ) \sim 1/{\ell }^{3}$. This behavior, which can be justified using perturbative arguments^4^, is at odds with known results for insulating many-body phases with short-range hopping^1^, indicating that other physical properties may also be unconventional. We thus proceed with analyzing the optical conductivity of the non-superfluid phase at *W* > *W*_c_.

The optical conductivity *σ*(*ω*) relates the current density *J* to the strength of an externally applied electric field ${{{{{{{\mathcal{E}}}}}}}}$ as $J(\omega )=\sigma (\omega ){{{{{{{\mathcal{E}}}}}}}}(\omega )$, with *ω* the field frequency. We obtain the optical conductivity *σ*(*ω*) within the linear response theory by first computing the current–current correlation function $\chi (\imath {\omega }_{n})={\langle j(\tau )j(0)\rangle }_{\imath {\omega }_{n}}/L$ at Matsubara frequencies *ω*_*n*_ = 2*πnT* using the Worm algorithm, followed by its numerical analytic continuation (see Methods). Here *j* is the lattice current operator defined as $j=\imath t{\sum }_{i\,{ < }\,j}{r}_{ij}[{b}_{i}^{{{{\dagger}}} }{b}_{j}-{b}_{j}^{{{{\dagger}}} }{b}_{i}]/{r}_{ij}^{3}$.

**Fig. 3:**
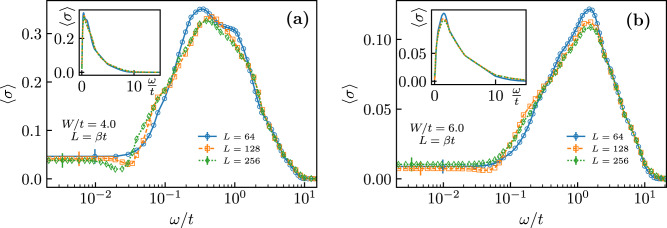
Disorder-averaged optical conductivity. 〈*σ*(*ω*)〉 as a function of the frequency *ω* for **a** *W* = 4 and **b** *W* = 6, and for system sizes *L* = 64 (blue circles), 128 (orange squares), and 256 (green diamonds). Data in the main plots were shown on the logarithmic scale for the frequency, highlighting the behavior at small *ω*. Insets show data on a linear scale. Vertical error bars indicate uncertainty of the extracted optical conductivity as determined by the analytic continuation procedure.

Figure 3 shows typical examples of the optical conductivity, averaged over a minimum of 384 disorder realizations, as a function of frequency for two values of *W* > *W*_c_ deep in the non-superfluid phase and different lattice sizes *L*. Consistently with the absence of superfluidity, the figure shows that the characteristic *δ*-functional peak at zero frequency peak in *σ*(*ω* ≃ 0) is absent. However, the numerical results also show two striking features: (i) The zero-frequency response is finite and system size-independent within the (relatively large) error bars; (ii) Unlike in usual conductors featuring a Drude peak (maximum at *ω* = 0), the optical conductivity has a minimum at zero frequency followed by a large peak at *ω* ≃ *t*, indicating strong response at energies of the order of the nearest-neighbor hopping amplitude. This peak broadens with increasing *W*, providing a large response up to frequencies *ω* ≃ 10*t*. Our results for the averaged conductivity demonstrate the existence of a conducting, non-superfluid phase of bosons in the ground state. This conducting behavior is not due to well-defined delocalized quasiparticle states as in a typical Drude-type metal; rather, it is an “anti-Drude metal”, where the largest response occurs at a small but finite frequency.

**Fig. 4:**
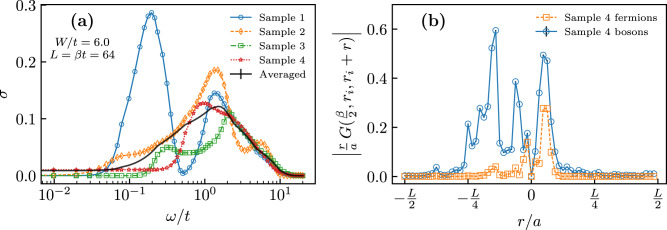
Overview of individual disorder realizations. **a** Optical conductivity as a function of frequency for different disorder realizations (the black continuous line is the average over all 384 disordered samples). **b** Correlation function $| r\,{{{{{{{{\mathcal{G}}}}}}}}}_{\tau = \frac{\beta }{2}}({r}_{{{{{{{{\rm{i}}}}}}}}},{r}_{{{{{{{{\rm{i}}}}}}}}}+r)|$ dependence on distance *r* with *r*_i_ being the location of the ${{{{{{{{\mathcal{G}}}}}}}}}_{\frac{\beta }{2}}({r}_{i},{r}_{i})$ maximum. Data were reported for both the bosonic system (blue circles) and the corresponding system of free fermions (orange squares). This plot visualizes dominant contributions to the current for a single disorder realization when the particle starts from point *r*_*i*_ (see main text and Methods). In both panels, data were shown for *L* = *β* = 64 and *W* = 6. Vertical error bars in **b** indicate the estimated uncertainty extracted from the Monte Carlo simulations.

Figure 4a shows selected results for *σ*(*ω*) in the aDMB phase for individual realizations of disorder, i.e., without averaging. We find that at frequencies *ω* > *t* the optical conductivity behavior is rather robust and sample-to-sample fluctuations are not substantial. The same cannot be said about the low-frequency part that wildly fluctuates from sample to sample—whilst some of the samples are metallic, the majority display an insulating behavior (the data for individual realizations also have at least an order of magnitude larger error bars, making low-frequency results for *σ*(*ω*) less reliable). This suggests that static *σ* might not be a self-averaging quantity in our system. These fluctuations will be also reflected in similar fluctuations in experiments.

The discovery of the aDMB phase is particularly surprising as the dipolar hopping term in Eq. (1) is usually considered to be short-ranged in one dimension. Nevertheless, it leads to large delocalized contributions to the current that can be visualized as follows. The single-particle propagator ${{{{{{{{\mathcal{G}}}}}}}}}_{\tau }(r,r^{\prime} )=\langle {b}_{r^{\prime} }^{{{{\dagger}}} }(\tau ){b}_{r}(0)\rangle$ encodes information for where a particle/hole injected into the system at site *r* can go in time *τ* (for hard-core bosons points *r* and $r^{\prime}$ are connected by a trajectory). By setting *τ* = *β*/2 and taking the limit *β* → *∞* we gain insight into the properties of the ground-state wave function. Since the current operator between distant sites involves an additional power of distance we multiply ${{{{{{{{\mathcal{G}}}}}}}}}_{\frac{\beta }{2}}(r,r^{\prime} )$ by $| r-r^{\prime} |$ to establish a quantitative measure for current contributions. Figure. 4b visualizes these contributions for a single conducting realization when the initial point *r*_*i*_ is chosen from the condition of maximum for ${{{{{{{{\mathcal{G}}}}}}}}}_{\frac{\beta }{2}}({r}_{i},{r}_{i})$. Data for the corresponding system of free fermions is presented alongside the bosonic case for comparison. The figure makes it clear that large current contributions are present over a wide range of distances of the order of ~*L*/4.

In summary, we have demonstrated that bosonic particles can exist in an unusual metallic phase at zero temperature. It emerges from the interplay between disorder, interactions, and dipolar hopping that have already been realized in experiments with Rydberg atoms, cold ions, and polar molecules. These results open many new research directions. These include investigations of other metallic phases that can exist in higher dimensions and possible connections to the experimentally observed “bad metal” states on the finite-temperature phase diagram of high-temperature superconductors.

## Methods

We perform quantum Monte Carlo simulations of Hamiltonian Eq. (1) in the path-integral representation in the grand-canonical ensemble using the worm algorithm^36^ for system sizes as large as *L* = 256 and temperatures as low as *T*/*t* = 1/256. At half-filling, we shift disorder realizations to ensue that 〈*W*_*i*_〉 = *μ* = 0 for each realization, with *μ* the chemical potential. The resulting density is then $\langle \rho \rangle =\frac{1}{2}$ when averaged over the disorder realizations with tiny, i.e. 2.8% for *L* = 256 and *W* = 6.0, sample-to-sample fluctuations.

In the presence of a constant vector potential Eq. (1) is modified by phase factors in the hopping elements of the form ${t}_{ij}\to {{{{{{{{\rm{e}}}}}}}}}^{\imath \phi {r}_{ij}}$. An expansion of the phase factor up to the second-order in *ϕ* leads to the current operator for the studied Hamiltonian

2 $$j=\imath t\mathop{\sum}\limits_{i < j}\frac{{r}_{ij}}{| {r}_{ij}{| }^{3}}\left[{b}_{i}^{{{{\dagger}}} }{b}_{j}-{b}_{j}^{{{{\dagger}}} }{b}_{i}\right]$$

along with the addition operator ${{{{{{{\mathcal{T}}}}}}}}$ that is required for a proper definition of the current–current correlation function (see below)

3 $${{{{{{{\mathcal{T}}}}}}}}=-t\mathop{\sum}\limits_{i < j}\frac{{r}_{ij}^{2}}{| {r}_{ij}{| }^{3}}\left[{b}_{i}^{{{{\dagger}}} }{b}_{j}+{b}_{j}^{{{{\dagger}}} }{b}_{i}\right].$$

The superfluid stiffness is, as usual, defined as the response of the free energy *F* to a weak externally applied phase *ϕ*

4 $${{{\Upsilon }}}_{s}=L\left.\frac{{\partial}^{2}F(\phi )}{\phi} \right|_{\phi = 0},$$

which in quantum Monte Carlo calculations is directly computed as^37,38^

5 $${{{\Upsilon }}}_{s}=\frac{L}{\beta }\langle {{{{{{{{\mathcal{W}}}}}}}}}^{2}\rangle ,$$

with ${{{{{{{\mathcal{W}}}}}}}}$ the path winding number. In the case of hopping connecting distant sites, as in Eq. (1), ${{{{{{{\mathcal{W}}}}}}}}$ can be written as ${{{{{{{\mathcal{W}}}}}}}}={N}_{\to }-{N}_{\leftarrow }={\sum }_{k}{r}_{k}$ with *N*_⇆_ the number of particle trajectories crossing the hypothetical boundary of the system in a given direction, and with the sum going over all the hopping elements in a single worldline configuration of the entire system (here, *r*_*k*_ represents the displacement between the sites connected by the *k*-th hopping event).

### Current–current correlation functions

In the regime of weak field *ϕ* (linear response) it is sufficient to look at the current–current correlation function

6 $$\chi (\imath {\omega }_{n})=\frac{{\langle j(\tau )j(0)\rangle }_{\imath {\omega }_{n}}}{L}$$

at Matsubara frequencies *ω*_*n*_ = 2*πTn* (*n* > 0). We compute it numerically and perform an analytic continuation procedure to obtain the conductivity *σ*(*ω*). Here, the subscript ı*ω*_*n*_ denotes that the Fourier transform is taken of the corresponding correlation function 〈*j*(*τ*)*j*(0)〉 in imaginary time.

Path-integral representation of quantum statistics for the Hamiltonian Eq. (1) allows one to sample Fourier components of this correlation function directly, and collect statistics for different Matsubara frequencies by using the estimator $| {\sum }_{k}\imath {r}_{k}{{{{{{{{\rm{e}}}}}}}}}^{\imath {\omega }_{n}{\tau }_{k}}{| }^{2}$, where again the sum goes over all hopping transitions on the system’s worldlines. For zero frequency *ω*_*n*_ = 0, this estimator is equivalent to measuring the winding number squared ${{{{{{{{\mathcal{W}}}}}}}}}^{2}$, while for large Matsubara frequencies it approaches the constant value corresponding to the estimator for ${{{{{{{\mathcal{T}}}}}}}}$. After computing statistical averages, we subtract $\langle {{{{{{{\mathcal{T}}}}}}}}\rangle$ from the data to obtain the current–current correlation function. To suppress finite-size effects associated with rare configurations with finite winding numbers, we restrict the sampling of the correlation function *χ*(*ıω*_*n*_) to configurations ${{{{{{{\mathcal{W}}}}}}}}=0$.

### Analytic continuation

Here, we are interested in computing the optical conductivity *σ*(*ω*), an observable that can be measured experimentally but is not readily accessible by numerical techniques. By the dissipation-fluctuation theorem,

7 $$\chi (\imath {\omega }_{n})=-\frac{2}{\pi }\int\nolimits_{0}^{\infty }\frac{{\omega }^{2}}{{\omega }_{n}^{2}+{\omega }^{2}}\sigma (\omega ){{{{{{{\rm{d}}}}}}}}\omega .$$

Finding *σ*(*ω*) is thus a standard ill-conditioned inverse problem when small fluctuations of the input due to statistical noise in the Monte Carlo sampling lead to large fluctuations in the output results. To solve this problem we use a method of consistent constraints^39,40^. It allows us to restore the spectral density *σ*(*ω*) from the corresponding correlation function *χ*(*ıω*_*n*_).

**Fig. 5:**
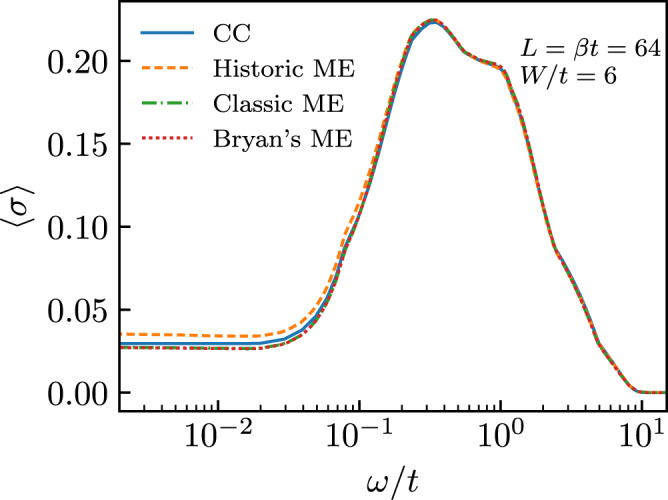
Comparison of analytic continuation algorithms on disorder-averaged data. Disorder-averaged optical conductivity 〈*σ*(*ω*)〉 as a function of the frequency for different analytic continuation algorithms including, consistent constraints (solid blue), and three different variants of the maximum entropy method: historic (dashed yellow), classic (dash-dotted green), and Bryan’s method (dotted red) [see ref. ^41^]. Data were shown for *L* = *β* = 64 and *W* = 6. The average is taken over all 384 disordered samples.

**Fig. 6:**
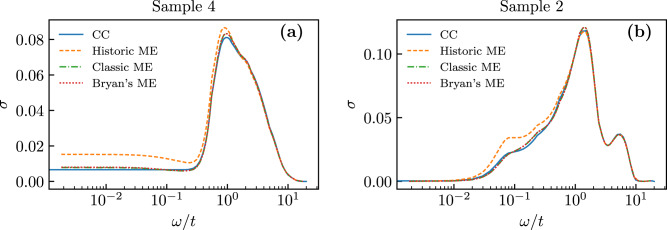
Comparison of different analytic continuation algorithms for individual disorder realizations. Optical conductivity *σ*(*ω*) in a system with *L* = 64, *β* = 64, and *W* = 6. Panels **a** and **b** correspond each to a different disorder realization. Different lines correspond to different algorithms including, consistent constraints (solid blue), and three different maxent variants: historic (dashed yellow), classic (dash-dotted green), and Bryan’s method (dotted red) [see ref. ^41^].

As a consistency check, we compare our results for the analytic continuation of our data with a standard implementation^41^ of the maximum entropy method^42^. We note that maxent suffers from numerical instabilities due to small errors on our data in the Matsubara frequency domain and it is able to find acceptable solutions only when artificially increasing the errors and using the solutions found with the method of consistent constraints as the “default model”. The comparison is shown in Fig. 5 in the case of the disorder-averaged conductivity and in Fig. 6 for single disorder realizations. Here, three different solutions are shown for maxent (ME), corresponding to the three different variations of the maximum entropy method available in the implementation of ref. ^41^ (historic, classic, and Bryan’s method). We see that, with the exception of the historic variant, all the solutions are essentially identical to each other and our solution is accepted by maxent with little or no modifications.

### Conductivity in the nearest-neighbors hopping model

**Fig. 7:**
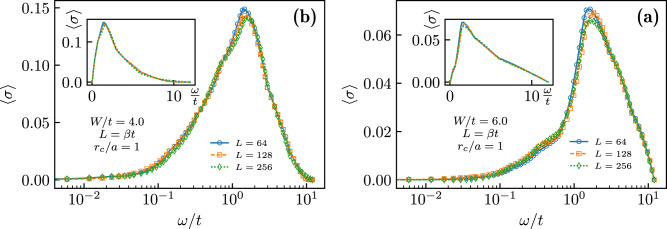
Optical conductivity for the nearest-neighbor hopping case. Disorder-averaged optical conductivity 〈*σ*(*ω*)〉 as a function of frequency *ω* for **a** *W* = 4 and **b** *W* = 6, for a system with nearest-neighbor only hopping. Data were shown for system sizes *L* = 64 (blue circles), 128 (orange squares), and 256 (green diamonds). Main plots show data on the logarithmic scale for the frequency, highlighting the behavior for small *ω*. Insets show data on a linear scale.

As a further check for the consistency of the analytic continuation procedure, in Fig. 7 we show the conductivity in the ground state of a system with short-range, nearest-neighbors hopping in the presence of disorder described by the Hamiltonian

8 $${{{{{{{\mathcal{H}}}}}}}}=-t\mathop{\sum}\limits_{\langle i,j\rangle }\left[{b}_{i}^{{{{\dagger}}} }{b}_{j}+{{{{{{{\rm{H.c.}}}}}}}}\right]+\mathop{\sum}\limits_{i}{\epsilon }_{i}{n}_{i}.$$

For these models, due to the phenomenon of Anderson localization^3^, we expect that the system is insulating at every finite disorder strength. From Fig. 7, it is clear that our method reproduces the expected results, with *σ*(0) = 0, for example, disorder strengths *W*/*t* = 4, 6.

### Disorder-averaged single-particle propagator

**Fig. 8:**
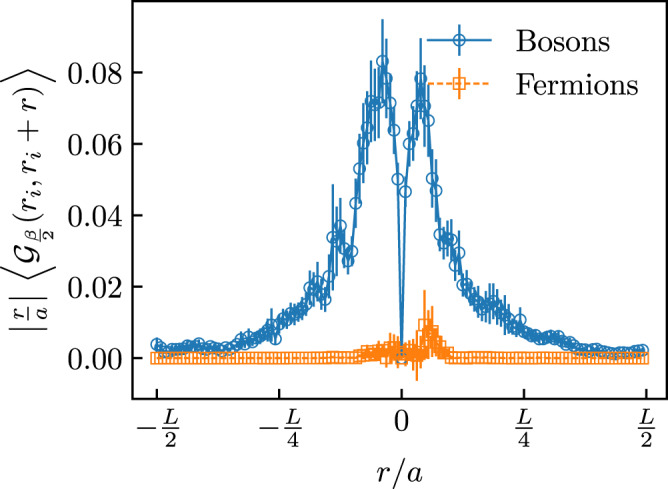
Disorder-averaged single-particle propagator. Disorder-averaged correlations $\langle | r{{{{{{{{\mathcal{G}}}}}}}}}_{\tau = \frac{\beta }{2}}({r}_{i},{r}_{i}+r)| \rangle$ as functions of the distance *r* for interacting bosons (blue circles) and the free fermion (orange squares) systems. Here, *r*_*i*_ is chosen so that, for each realization of the disorder, the bosonic ${{{{{{{{\mathcal{G}}}}}}}}}_{\tau = \frac{\beta }{2}}({r}_{i},{r}_{i})$ is at its maximum. Data were shown for *L* = *β* = 128 and *W* = 6 and the average is carried over 128 disorder realizations. Vertical error bars indicate the estimated uncertainty extracted from the Monte Carlo simulations and averages over disorder.

The quantity $| r-r^{\prime} | {{{{{{{{\mathcal{G}}}}}}}}}_{\frac{\beta }{2}}(r,r^{\prime} )$, as shown in Fig. 4b of the main text, constitutes a qualitative measure for current contributions and of long-range coherence in the system. Figure. 8 shows the same quantity averaged over 128 disorder realizations for systems with *L* = *β* = 128 and *W* = 6. The average is carried by choosing, in each realization, the initial point *r*_*i*_ for which ${{{{{{{{\mathcal{G}}}}}}}}}_{\frac{\beta }{2}}({r}_{i},{r}_{i})$ is maximum. The figure shows clearly, also by direct comparison with the same quantity computed for a system of non-interacting fermions, that in the disorder-averaged picture bosons have much larger current contributions, especially at large distances.

## Data Availability

The data that support the findings of this study are available from the corresponding author upon reasonable request.

## Code availability

The code for the numerical simulations is available from the corresponding author upon reasonable request.
